# The Role of Oxidative Stress in Multiple Exercise-Regulated Bone Homeostasis

**DOI:** 10.14336/AD.2023.0223

**Published:** 2023-10-01

**Authors:** Haoyang Gao, Yilong Zhao, Linlin Zhao, Zhikun Wang, Kai Yan, Bo Gao, Lingli Zhang

**Affiliations:** ^1^School of Exercise and Health, Shanghai University of Sport, Shanghai, China; ^2^Putuo Hospital, Shanghai University of Traditional Chinese Medicine, Shanghai, China; ^3^Institute of Orthopedic Surgery, Xijing Hospital, Fourth Military Medical University, Xi’an, China; ^4^College of Athletic Performance, Shanghai University of Sport, Shanghai, China

**Keywords:** exercise type, endurance sports, resistance training, speed training, impact training, flexibility training, bone remodeling, oxidative stress

## Abstract

Bone is a tissue that is active throughout the lifespan, and its physiological activities, such as growth, development, absorption, and formation, are always ongoing. All types of stimulation that occur in sports play an important role in regulating the physiological activities of bone. Here, we track the latest research progress locally and abroad, summarize the recent, relevant research results, and systematically summarize the effects of different types of exercise on bone mass, bone strength and bone metabolism. We found that different types of exercise have different effects on bone health due to their unique technical characteristics. Oxidative stress is an important mechanism mediating the exercise regulation of bone homeostasis. Excessive high-intensity exercise does not benefit bone health but induces a high level of oxidative stress in the body, which has a negative impact on bone tissue. Regular moderate exercise can improve the body's antioxidant defense ability, inhibit an excessive oxidative stress response, promote the positive balance of bone metabolism, delay age-related bone loss and deterioration of bone microstructures and have a prevention and treatment effect on osteoporosis caused by many factors. Based on the above findings, we provide evidence for the role of exercise in the prevention and treatment of bone diseases. This study provides a systematic basis for clinicians and professionals to reasonably formulate exercise prescriptions and provides exercise guidance for patients and the general public. This study also provides a reference for follow-up research.

## 1. Introduction

Physical activity occurs throughout the lifespan, and as a very common physiological stimulus, it can cause homeostatic and metabolic changes in the body [1]. Long-term and sustained exercise will cause corresponding adaptive changes in the morphology and function of tissues and organs and ultimately have a profound effect on the body [2].

Bone is one of the basic components of the human motion system, and is involved in basic functions including movement, support and protection, and hematopoiesis [3]. As an active tissue with important physiological functions, the metabolic activity of bone runs through the entire life cycle of an individual until death [4]. During this process, bones undergo various physiological changes, such as formation, growth, absorption and remodeling [4]. These dynamic changes are affected by a variety of internal and external environmental factors, such as immunity [5], endocrine activity [6] and nutrition [7]. As early as 1892, the famous surgeon Wolff proposed a law stating that the growth, absorption and reconstruction of bones are related to the stress placed on the bones[8]. This law reveals the close relationship between bone tissue and mechanical load. Later, Frost[9] further proposed the concept of a mechanical regulation system (mechanostat) and stated that bone growth and loss of bone mass can be regulated by the local mechanical and elastic deformation of bone. That is, bone remodeling can be regulated by local mechanical stimulation of bone, thus providing important insight for subsequent research.

The effects of physical activity on bones have also been demonstrated in numerous studies throughout the ages. Exercise has an important influence on bone quality, morphological structures, mechanical properties and physiological properties[10]. Different types of exercise, due to their unique technical characteristics and force application methods, have different effects on bones[11]. One can intentionally promote bone development in the desired direction by choosing to participate in the appropriate type of exercise. The prevention and treatment of osteoporosis (OP)[12], osteoarthritis (OA)[13], and other orthopedic illnesses, as well as the promotion of bone health, have drawn significant attention to exercise as a cost-efficient, safe, and effective health intervention approach.

Estrogens and androgens affect bone growth and maintenance. They promote bone mass gain during puberty, whereas decreased estrogen levels in menopausal women or concurrent loss of estrogen and androgens in older men can lead to decreased bone mass and strength, leading to the development of OP[14, 15]. Sex hormones play an important role in maintaining bone mass and strength in adults by slowing bone remodeling and maintaining the balance between resorption and formation[15]. However, at the same time, some studies have also revealed the negative regulation of sex hormones on bone metabolism in some cases. Estrogen can indirectly negatively regulate bone homeostasis through central nervous system pathways[16]. Androgens appear to inhibit load-induced bone formation by inhibiting Wnt/β-catenin signaling secondary to upregulated sclerostin (SOST)[17](Table 1).

**Table 1:** Effect of sex hormones on bone metabolism.

Sex-related hormones	Positive effects	Negative effects
Estrogens	Inhibit osteoclast formation and promote osteoclast apoptosis; inhibition of apoptosis in osteoblasts and osteocytes [14, 15]	Mediate negative central nervous system effects on bone [16]
Androgens	Inhibit osteoclast formation and promote osteoclast apoptosis; inhibition of apoptosis in osteoblasts and osteocytes [14, 15]	Inhibition of mechanical load-induced osteogenesis [17]

## 2. Endurance exercise and bone

**Figure 1.**
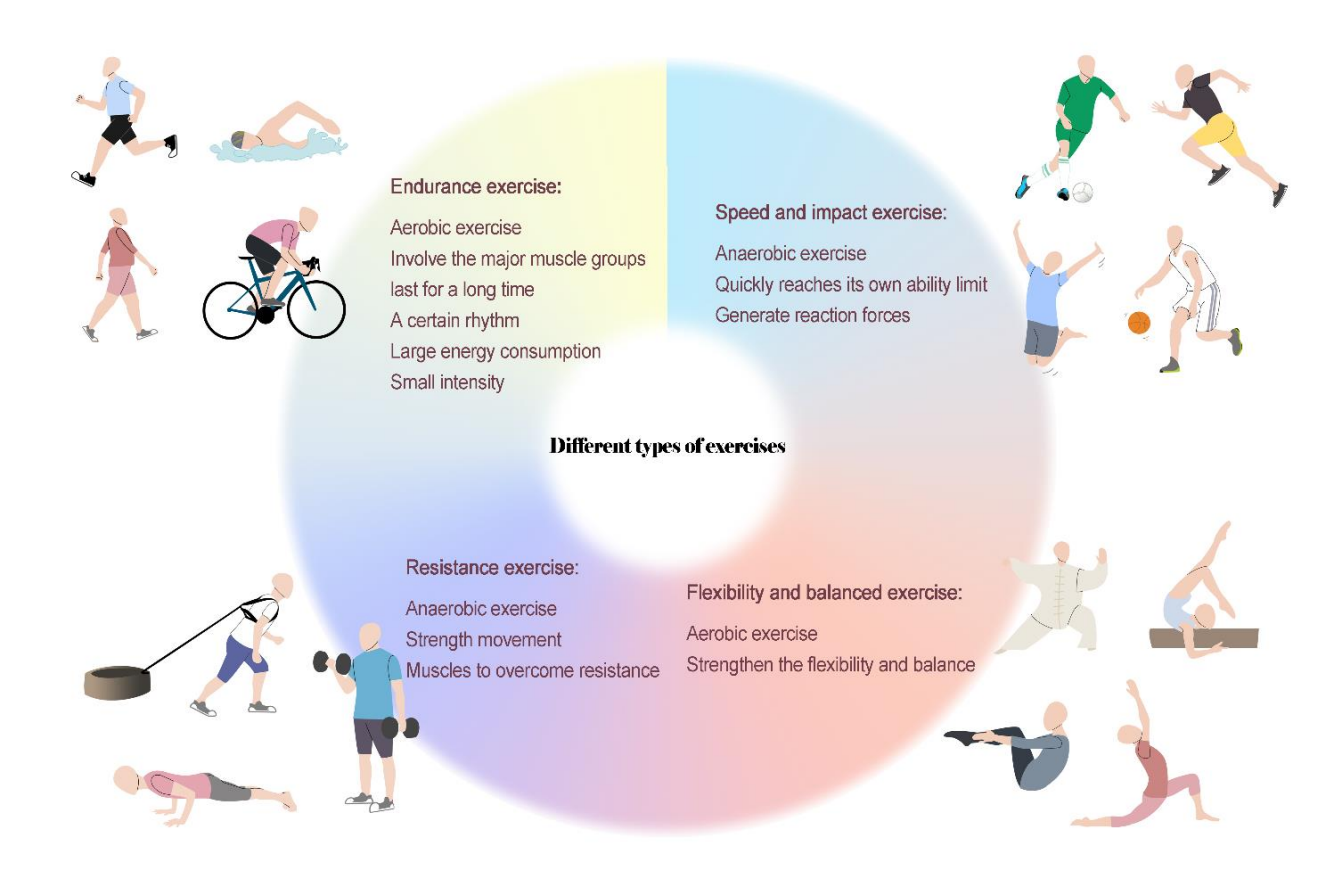
Definition and characteristics of different types of exercises.

Endurance exercise is the simplest and most common form of physical exercise in daily life and the most basic and main exercise means in the medical exercise prescription. The major muscle groups of the body are typically used in endurance exercise, which is long-lasting and rhythmic. Endurance exercise is characterized by a lengthy activity period, high energy expenditure, and a lack of rest periods, although the exercise intensity is relatively low. Since the majority of the energy required for exercise comes from the aerobic (oxidative) energy system and since the relationship between exercise load and oxygen consumption is linear, endurance exercise is classified as aerobic exercise from the standpoint of energy metabolism. Moderate- or high-intensity endurance exercise is an effective means to improve cardiopulmonary function, prevent and treat chronic metabolic diseases and regulate the psychological and mental state. Currently, the main forms of endurance exercise include jogging, walking briskly, cycling and swimming (Fig. 1).

### 2.1 Effects of endurance exercise on bone mass

Bone mineral density (BMD) and bone mineral content (BMC) are the criteria reflecting bone mass and are used for the diagnosis of OP and in the risk assessment of fracture. Currently, dual-energy X-ray absorptiometry (DEXA) is considered the gold standard for BMD. Bone microstructures can be detected using bone histo-morphometry or a micro-CT scanning system, especially the latter, which can establish a more intuitive three-dimensional image with the help of a computer system. With micro-CT scanning, the trabecular volume (BV/TV), trabecular number (Tb. N), trabecular separation (Tb. Sp), trabecular thickness (Tb. Th), structure model index (SMI) and many other bone analysis parameters can be determined. There are many factors contributing to a decline in BMD, including aging, hormones, a microgravity environment (such as space flight), and fractures. Persistent decreases in BMD without controlled intervention will lead to OP, in turn increasing the risk of fracture. OP caused by decreased BMD has been shown to account for approximately half of hip fractures[18]. Currently, the mainstream methods for improving BMD include medication, nutrition interventions, and exercise therapy, although commonly used clinical drugs (such as bisphosphate and teriparatide) have also shown good therapeutic effects and can reduce the risk of fracture caused by decreased BMD by approximately 20% to 60% [19]. However, drug treatment also often has many shortcomings, such as complications caused by long-term use, poor patient compliance and low durability [20, 21]. Additionally, other important fracture risk indicators such as muscular strength, dynamic balance, coordination, or overall fitness level do not improve with treatment [22]. Exercise is a strategy that can improve all of the controllable underlying fracture risk factors (bone strength, risk of falling, and impact of falling) [22]. In evidence-based medical research, there is grade A (strong) evidence that physical activity can improve BMD, and the effect of physical activity on bone structure is supported grade B (medium strength) evidence [23].

### 2.1.1 Effects of endurance exercise on the bone mass of children and adolescents

Proper exercise is of great significance for the growth, development and bone health of preschool children and adolescents. Teenagers are in a critical stage of body growth and development. Their bone tissues are characterized by good toughness and strong plasticity and are easily affected by external factors, such as diet, exercise, and posture. Bone health in adulthood and middle and old age is closely related to bone development in adolescence. Childhood and adolescence are periods of rapid BMD growth, which are critical for both lifetime bone mass and BMD [24]. It has been reported that approximately 94% of a person's lifetime peak bone mass (PBM) is obtained before the age of 16 years old [25]. Epidemiological studies have shown that a 10% increase in PBM in a population reduces the risk of fractures later in life by 50% [26]. Therefore, the promotion of PBM through exercise in childhood and adolescence has become one of the most commonly used methods to prevent future OP and reduce fracture risk[27, 28]. Although the importance of exercise for young children is increasingly emerging, the results of most current studies have shown that endurance sports, such as jogging and swimming, do not seem to be the optimal choice to promote children's bone growth and development. For example, although swimming has health benefits, such as improving cardiopulmonary function and increasing muscle strength, a study showed that regular swimming did not cause significant improvement in whole body, femoral neck or lumbar vertebral BMD, or in other BMD sites in teenagers, children and young individuals[29, 30]. This finding might have occurred because swimming in water does not provide sufficient mechanical load stimulation to the bones. Similarly, a cross-sectional study of female high school distance runners aged approximately 15 years old showed that the more advanced the athlete was, the greater the likelihood of low BMD [31]. This result further indicates that long-term endurance exercise is not conducive to adolescent BMD and might even have a negative impact.

### 2.1.2 Effects of endurance exercise on the bone mass of middle-aged and elderly individuals

OP is a major health concern for the aging population, and in the United States, approximately 54% of the population aged 50 and older has OP with a decrease in BMD [32]. Although physiological aging, body weight loss, and nutrition are the main factors related to the progressive decline in BMD in elderly individuals, the incidence rate of OP is significantly different between the sexes due to sex hormones. Women, especially postmenopausal middle-aged and elderly women, have relatively poorly bone microstructure and BMD levels and a high risk of fracture [33, 34]. The decline in estrogen levels after menopause is one of the important reasons for the decline in BMD in middle-aged and elderly women. Fortunately, studies have found that, compared with men, the effect of exercise on improving the bone health of women is more significant: among aged women who often participate in exercise during adolescence and old age, the lumbar BMD is higher, and their risk of OP is lower [35]. Therefore, elderly individuals, especially women, should engage in resistance training for muscular strength to maintain a good BMD condition before menopause to delay the onset of the physiological factors associated with the aging-related decline in BMD. However, due to body weight reductions and other reasons in elderly individuals, the bone load force from simple physical activity is not sufficient to achieve the required load threshold for bone movement and adaptation. Therefore, it has been demonstrated that low-intensity or impact-free endurance activities like cycling and swimming have no influence on postmenopausal women's ability to prevent age-related bone loss [22]. However, the cardiopulmonary function and muscle strength of elderly individuals restrict their ability to exercise with high intensity and a high load. Therefore, for the middle-aged and elderly population, the combination of endurance exercise with other types of exercise or other interventions, such as physical therapy, is often used to improve BMD and BMC. Eid et al.[36] significantly improved the BMD levels of female OP patients by combining pulse magnetic therapy with moderate intensity endurance exercise. Zhao et al. [37] conducted a meta-analysis of the prevention effect of combined exercise intervention, including endurance exercise, on bone loss in middle-aged and elderly postmenopausal women, and the results showed that a combined exercise intervention can effectively maintain the BMD level of the lumbar spine, femoral neck, whole hip and whole body in postmenopausal women, and the therapeutic evidence level reached 1A.

In addition to clinical trials and randomized, controlled trials targeting specific populations, relevant animal research experiments have also been conducted. Gao et al. [38], using ovariectomized female rats to mimic menopausal women, showed that 17 weeks of moderate intensity running significantly improved the ovariectomized-induced decreases in BMD and negative changes in trabecular bone morphology. Similarly, Sato et al. [39] demonstrated that 12 m/min endurance running increased the percentage of BMD, bone strength, and skeletal muscle mass in ovariectomized and tail-suspended rats and decreased the body fat percentage.

### 2.1.3 Effects of endurance exercise on bone mass in special populations

In addition to the decline in BMD caused by the physiological factors mentioned above, OP is also a common complication of AIDS, cancer, type II diabetes, etc. Exercise training similar to that in the elderly can also improve the bone loss caused by these specific pathological factors. A randomized, controlled trial showed that a combination of six months of resistance and endurance exercise selectively improved lumbar spine and femoral neck BMD and the grip strength in HIV-infected patients without any sex differences [40]. In cancer patients, a mixed exercise mode can also lead to increases in whole-body and multi-site BMD [41].

Based on traditional thinking, professional athletes should be much better than the general population with respect to cardiopulmonary function, strength, BMD and other physical qualities. However, a survey of professional athletes and high-level amateurs found that this assumption was not true. Hind et al. [42] found that the lumbar BMD of endurance distance runners was relatively low, and the degree of decline was positively correlated with the running distance per week and the training year, suggesting that distance runners might be at risk of poor health of the lumbar spine bone. Bellver et al. [43] concluded that the BMD and BMC of female athletes in aquatic sports (swimming, synchronized swimming, etc.) were significantly lower than those in nonaquatic sports (football, volleyball, etc.) and were even comparable to the BMD of the sedentary population. Due to the characteristics of a long exercise duration and high amount of single exercise in endurance sports, overtraining is likely to occur, which can lead to the adverse consequences of large amounts of trace element loss, electrolyte and endocrine disorders in athletes, and estrogen decline in female athletes and menstrual disorders, which are the potential factors leading to bone loss and decreased BMD and BMC levels. Therefore, specific athletes engaged in long-distance running, swimming and other endurance sports should also include weight bearing or strength training exercise in their training routine to ensure bone health.

In contrast, the results of multiple rodent-based studies have been different from those of human studies. Chen et al. [44] showed that endurance running could improve the BMD of mice and that nuclear factor erythroid derived 2-related factor 2 (Nrf2) mediated the protection of bone by exercise. In addition, autonomic rotation partially reversed the bone loss caused by a high-fat diet and increased BMD [45, 46]. It also positively improved BV/TV, Tb.Sp, Tb.Th and other indicators, and had favorable effects on bone microstructure [45, 46].

**Figure 2.**
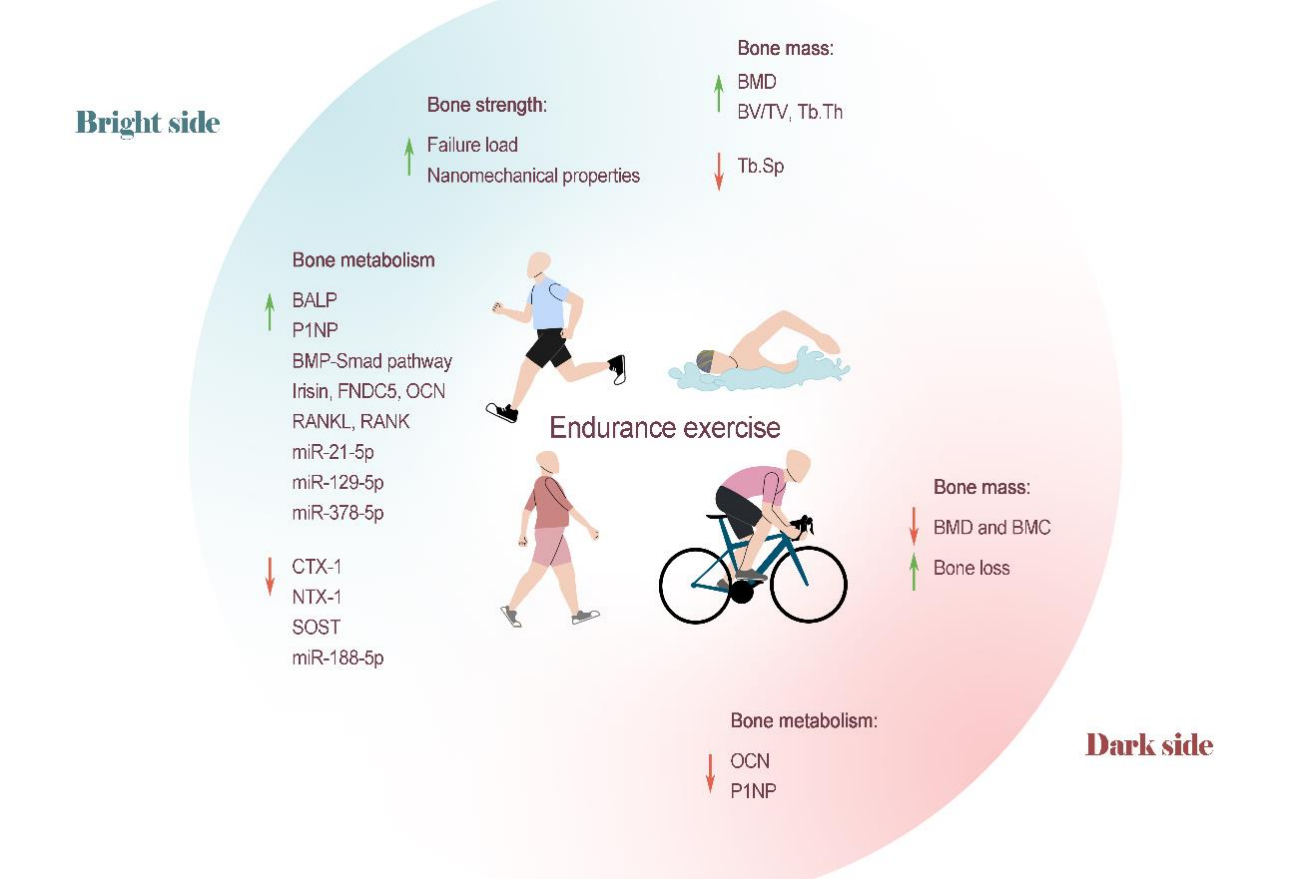
Endurance exercise on bone health. The majority of human studies have found that pure endurance training does not exert beneficial effects related to increasing bone mass or improving BMD and BMC and even leads to bone loss and decreases in BMD and BMC. Therefore, endurance exercise alone is not recommended as the preferred type of exercise for children, teenagers, middle-aged individuals and elderly individuals who must increase or improve bone mass. However, some animal experimental reports have reached different conclusions. Most studies have shown that endurance exercise can promote bone formation and inhibit bone resorption. Although endurance exercise has shown a trend toward improvement and the potential to improve bone mechanical strength, it is difficult to find significant differences in statistical calculations.

In summary, the vast majority of human studies have found that pure endurance training does not exert positive effects on increasing bone mass and improving BMD and BMC and is even accompanied by adverse risks such as excessive exercise. Therefore, single endurance exercise is not recommended as the preferred type of exercise for children, teenagers, middle-aged individuals and elderly individuals who must increase or improve their bone mass. However, some animal experimental reports have held different conclusions. Therefore, the key to the positive regulation of exercise regarding bones is that the bones receive high mechanical loads rather than long times of activity (Fig. 2).

### 2.2 Effects of endurance exercise on bone strength

Bone biomechanical properties comprehensively reflect the mechanical strength and morphology of bone and directly reflect the intrinsic properties and quality of bone. The most popular technique for determining the maximum load, yield stress, elastic modulus, and stiffness of bone is three-point bending. Bone mass and bone quality make up the majority of bone strength. Although bone mass determines approximately 80% of the biomechanical strength of bone, data from recent studies have indicated that BMD does not completely stipulate the biomechanical parameters [47]. These are related to the internal structure of bone, such as the three-dimensional architecture of bone mass and the connectivity between trabeculae, in addition to the bone mass. At the same time, Frost's research showed that the effects of hormones, calcium and vitamin D on bone strength are only 3% to 10%, while the mechanics are as high as 40%.

Some factors, such as ovariectomy [48] or obesity [45], can seriously damage the mechanical strength of bone. However, studies have shown that endurance exercise can improve bone biomechanical properties. Liu et al. [49] performed treadmill training in male Sprague-Dawley rats (SD rats) for 4 weeks and found that 12 m/min aerobic running exercise caused significant increases in failure load and significantly higher nanomechanical properties of cortical bone. In fact, we found that, although endurance exercise shows a trend toward bone improvement and has the potential to improve bone mechanical strength, it is difficult to achieve significant differences in statistical calculations[45]. On the one hand, bone biomechanical properties are relatively stable and not easily affected by external intervention; on the other hand, compared with other types of exercise, the mechanical stimulation that can be applied to bones by shorter endurance exercise is inherently weaker, and changes in bone mechanical properties are less likely to be captured (Fig. 2).

### 2.3 Effects of endurance exercise on bone metabolism

Bone tissue is a dynamic tissue composed of cells (osteoblasts, osteoclasts, and osteocytes) and extracellular matrix. Osteoblasts are derived from undifferentiated bone marrow mesenchymal stem cells, while the progenitor cells of osteoclasts are monocytes and macrophages. Bone metabolism (also known as bone remodeling or bone turnover) is a physiological process of continuous renewal of bone mineral content and bone matrix that includes two processes, namely, osteoclast-mediated bone resorption and osteoblast-mediated bone formation. The activity of bone metabolism is closely related to the activity of various bone cells. Under normal circumstances, the mass of old bones absorbed by osteoclasts is consistent with the mass of new bones generated by osteoblasts to realize the continuous renewal of bone tissues and their mechanical integrity in the processes of life, and to play an important role in maintaining the homeostasis of trace elements, such as calcium and phosphate, in the body. However, under some physiological or pathological conditions, the activities of osteoclasts and osteoblasts are changed, and the balance of bone metabolism is broken, leading to the physiological growth and development of bone tissue, as well as aging-related or pathological OP, bone-softening diseases, bone metastasis, etc.

The changes in bone metabolism will ultimately be manifested in indicators such as bone mass, bone morphological structure and biomechanical properties. However, traditional radiographic methods often have a certain lag in the measurement of BMD, making it difficult to achieve the early diagnosis and evaluation of abnormal conditions. Later, it was gradually discovered that some specific proteins and peptides, which are called bone turnover markers (BTMs), are released in the process of bone metabolism [50]. BTM levels are sensitive to changes in the bone turnover rate and have been used as early indicators for monitoring the treatment of bone metabolic disorders [51]. BTMs are divided into bone formation markers and bone resorption markers. Currently, commonly used bone formation markers include alkaline phosphatase (ALP), N-terminal peptide of type I collagen (PINP), and osteocalcin (OCN) [52]. Bone resorption markers include tartrate-resistant acid phosphatase-5b (TRAP-5b), carboxy-terminal cross-linked telopeptide of type 1 collagen (CTX-1), amino-terminal cross-linking telopeptide of type I collagen (NTX-1), hydroxyproline (HYP/HOP), and pyridinoline (PYD) [52]. Among these markers, serum PINP and CTX-1 have been recommended by the International Osteoporosis Foundation (IOF) and the International Federation of Clinical Chemistry (IFCC) as reference markers of bone formation and bone resorption for risk prediction and monitoring of fracture treatment for OP [53]. However, the levels of BTMs in animals are affected by a variety of factors, such as decreases in bone resorption markers that could be due to food intake; an increases in all marker levels, being due to an acute fracture response [54]; and the lack of sufficient evidence-based data to support a standard threshold for BTMs. Therefore, each BTM index cannot be used solely for the diagnosis of OP and prediction of fracture risk in the absence of other findings. Undeniably, the convenience, rapidity and relative noninvasiveness of BTM measurement have given it an irreplaceably important position in clinical and scientific research. In addition, bone histomorphometry is also an important means to investigate the state of bone metabolism with excellent reliability and validity. However, it is generally used for scientific research and is not a commonly used method in clinical practice due to its complex operation, strict requirements and trauma.

By detecting the level of BTMs in serum or urine, we can understand the metabolism of bone tissue, evaluate the state of bone metabolism, predict the risk of fracture, differentiate the diagnosis of metabolic bone disease, and detect the therapeutic effect.

### 2.3.1 Effects of endurance exercise on bone metabolism in children and adolescents

Physical activity in healthy prepubertal girls were positively correlated with BMD and serum bone-specific ALP (BALP) concentrations and negatively correlated with CTX-1 concentrations, suggesting that physical activity increased the BMD and BMC of prepubertal girls by promoting bone formation and inhibiting bone resorption [55]. As mentioned above, sports with relatively low loads, such as jogging, cycling and swimming, are generally considered to be lifetime fitness activities for the elderly, rather than bone-building activities. Although a large number of studies have confirmed the lack of positive effects of these endurance activities on BMD and BMC levels in adolescents, there is not much direct and strong evidence regarding the related BTM indicators. The research results of Rapun-Lopez et al. [56] showed that, with increasing age, OCN and PINP concentrations decreased in both the cycling group and the control group, while β-CTX concentrations showed no significant difference. However, it should be pointed out that, during this period, the vitamin D level of the cyclists decreased significantly. Combined with previously reported findings, the BMC and BMD of adolescent cyclists older than 17 years of age were significantly higher than those of controls [57], and it is currently uncertain whether changes in BTM accurately reflect changes in bone during adolescent growth and development. Additionally, Kim et al. [58] observed that, after 12 weeks of training, there was no specific significant difference in the concentrations or mRNA expression levels of receptor activator of nuclear factor-κB receptor activator ligand (RANKL) and osteoprotegerin (OPG) in the peripheral serum of young women before and after the training, suggesting that endurance exercise might have a limited regulatory effect on the RANKL/receptor activator of NF-κB (RANK)/OPG signaling pathway in adolescents.

Zhang et al. [59], using bone histomorphometry and measurement of the related BTM concentrations in peripheral circulation, found that five weeks of moderate intensity treadmill exercise significantly promoted bone formation and inhibited bone resorption in mice in the growth phase, thereby significantly improving BMD and bone mass. Further research suggested that moderate intensity running exercise might mediate the positive effects on bones by activating the bone morphogenic protein (BMP)-Smad signaling pathway in mice and promoting the phosphorylation of the Smad1 protein and the osteogenic differentiation of bone marrow mesenchymal stem cells [59]. In 2012, Bostrom et al. [60] found a new myogenic factor, irisin, which was produced by exercise induction and processed by proteolysis of the product of the fibronectin domain-containing 5 (FNDC5) gene. It promoted heat production and browning of white adipose tissue. It was later found that irisin could regulate bone metabolism and mediate movement-induced bone turnover. Multiple types of exercise can cause an increase in circulating irisin concentrations. Zhang et al. [61] demonstrated that two weeks of voluntary wheel running could induce high-level expression of FNDC5 mRNA and FNDC5/irisin protein in the bone tissue of 5-week-old young mice and could increase the expression of OCN and other factors. At the same time, in vitro experiments showed that irisin increased osteoblast formation and mineralization and inhibited RANKL-induced osteoclastogenesis [61]. This study confirmed that aerobic endurance exercise increases the production of irisin in bones and that the increased level of circulating irisin enhances the osteogenic ability of mice in the growth phase.

### 2.3.2 Effects of endurance exercise on bone metabolism in middle-aged and elderly individuals

Endurance exercise promotes a positive balance of bone metabolism in adult OP patients. Roghani et al. [62] found that after six weeks of aerobic exercise training, postmenopausal women with OP experienced significant increases in BALP concentrations, and significant decreases in NTX-1 concentrations, indicating that exercise training could stimulate bone synthesis and inhibit bone resorption in postmenopausal women with OP. Arazi et al. [63] conducted a randomized, controlled trial and reported that the combination of endurance and resistance training and milk supplementation could improve BMD and blood ALP levels in adult female OP patients. MicroRNAs (miRNAs) are a class of short-chain noncoding RNAs that regulate gene transcription and have been found to be important information carriers mediating exercise-induced osteogenesis. It has been reported in the literature that, after long-distance endurance running, the serum levels of miR-21-5p, miR-129-5p and miR-378-5p in adult men significantly increased, while the expression of miR-188-5p was downregulated[64]. The former three out of the four aforementioned miRNAs can activate the downstream signaling pathway and promote the differentiation of osteoblast precursor cells into osteoblasts, while the latter miRNA mediates the adipogenic differentiation of bone marrow mesenchymal stem cells, also demonstrating that miRNAs can be regulated by aerobic exercise, thus promoting the osteogenic differentiation of cells and inhibiting their adipogenic differentiation [64].

### 2.3.3 Effects of endurance exercise on bone metabolism in special populations

OP and increased fracture risk are common complications in patients with diabetes and often manifest as decreased BMD and decreased bone turnover. Physical activity has been proven to be a good choice to improve glycolipid metabolic disorders and bone health in patients with diabetes. Among many types of exercise, moderate-intensity, moderate-duration aerobic exercise is the most commonly recommended physical activity by the American Diabetes Association (ADA) [65]. The effect of aerobic endurance exercise on bone metabolism in patients with diabetes has also been frequently reported. Elhabashy et al. [66] reported that serum PINP concentrations of adolescents with type I diabetes were low, and after three months of regular aerobic endurance training, their serum PINP concentrations were significantly increased, accompanied by improvements in BMD. After a single aerobic exercise, the BTM index changes in patients with type I diabetes are similar to those in healthy people, suggesting that the response of patients with diabetes to exercise were normal to a large extent, thus providing the possibility of exercise therapy to improve the bone health of patients with diabetes [67]. In addition, although the serum β-CTX concentrations of the two groups were decreased, the decreased amplitude of serum β-CTX concentrations in patients with diabetes was significantly lower, also indicating that type I diabetes might limit the inhibitory effect of exercise on bone resorption and thus accelerate bone loss [67]. Decreased serum BTM levels and decreased bone turnover in patients with type I and type II diabetes are important features of their bone metabolism and factors contributing to an increased risk of bone fracture [68, 69]. Studies have asserted that exercise interventions can improve bone metabolism in patients with diabetes. Abildgaard et al. [70] reported that twelve months of an exercise intervention (a combination of endurance and resistance training) could significantly increase serum PINP, OCN, and CTX-1 concentrations in patients with type II diabetes, indicating effective improvements in bone turnover and bone health but with no significant changes in BMD.

Chronic kidney disease (CKD) can lead to metabolic disorders of hormones, electrolytes, and minerals, and it is a major pathological factor in secondary OP. Liao et al. [71] showed in animal experiments that, after eight weeks of endurance running, the serum concentrations of SOST and CTX-1 in CKD rats were significantly decreased, while the activity of Wnt/β-catenin, an osteogenic signaling pathway inhibited by SOST, was significantly increased. It was thus confirmed that endurance exercise ameliorated CKD-induced BMD and bone microarchitecture damage by inhibiting the production of SOST, but it had no effects on serum minerals. In addition to secondary OP, CKD can also cause bone developmental delays. However, endurance running can promote the bone growth and development of CKD rats in the growth phase and can increase the expression of RANKL in the epiphyseal growth plate, with a certain therapeutic effect [72]. Fluoride poisoning can lead to skeletal fluorosis, causing oxidative stress damage to bone tissue, in turn leading to osteomalacia or OP, thereby damaging bone tissue [73]. Li et al. [73] found that each mRNA and protein level in the OPG/RANKL/RANK signaling pathway was increased in fluoride-poisoned mice, which accelerated bone turnover and destroyed bone remodeling. Interestingly, exercise intervention did not directly increase OPG expression, and it might play a therapeutic role by eliminating excessive fluoride and indirectly regulating the OPG/RANKL/RANK signaling pathway [73].

In summary, endurance exercise can regulate bone metabolism by activating or inhibiting signaling pathways related to bone metabolism, inducing the release of related hormones and cytokines, and interfering with the expression of certain noncoding RNAs. New targets for the prevention and treatment of OP-related disorders have been made possible by these results. Currently, the majority of studies—whether conducted on animals or humans—have demonstrated that endurance exercise can encourage bone formation and inhibit bone resorption. However, in studies of children and adolescents, endurance exercise did not show the above effects and even led to a negative balance of bone metabolism in some cases (Fig. 2).

## 3. Resistance training and bone

Resistance training involves the active contraction of skeletal muscle to overcome resistance and create movement, and it is usually called strength training. Resistance training is often used to shape the body and restore and improve muscle strength, in addition to playing some role in improving both flexibility and balance. During strength training, resistance can be applied by others with their healthy limbs or with external loads/devices, and the amount of resistance needed depends on the muscular strength level of the participant. Typically, resistance training is mostly anaerobic exercise, but it can also combine the advantages of aerobic exercise by adopting the formula of light loads, multiple repetitions and multiple groups of exercises performed in a circuit. Currently, resistance training has been widely used in the clinical treatment of OP and various causes of muscle atrophy and has achieved significant results. In a broad sense, all types of exercise that exert additional loads on skeletal muscle can be called resistance training, and the common forms include weight-bearing exercise, push-ups, weight-lifting, and other exercises using various types of fitness equipment (Fig. 1).

### 3.1 Effects of resistance training on bone mass

### 3.1.1 Effects of resistance training on bone mass of children and adolescents

Regular physical exercise, especially weight-bearing resistance training, is conducive to bone mass accumulation and PBM acquisition [27]. In addition, vigorous physical activity is also beneficial to the mineral accumulation process of bone structures [27, 74, 75]. Studies have confirmed that both short-term and long-term resistance training can effectively promote the level of BMD and BMC and optimize the bone health of adolescent girls [76, 77]. In addition, resistance training affects the BMD of specific parts of girls in the growth phase, and a higher frequency of resistance training can significantly increase the BMD of their lumbar spine [78]. Young children who suffer from cerebral palsy, a neurological condition, may have cognitive and physical impairments. Due to their reduced mobility, inadequate nourishment, pharmacological side effects, and other circumstances, children with cerebral palsy are at a significantly higher risk of secondary OP and fractures than normal children. Exercises involving weight bearing have been researched and used to help children with cerebral palsy's bones. Kim et al. [79] reported that weight-bearing exercise was found to significantly improve the BMD of the femur but not of the lumbar spine in children with cerebral palsy. This outcome might have occurred because the femur is subjected to less mechanical force than the lumbar spine for children with cerebral palsy who have difficulty maintaining an upright position [79]. In animal models, load-bearing ladder climbing is the most commonly used method to simulate resistance training for rodents. Ladder-climbing training has had positive effects on the bones of many different rat models. Weight-bearing ladder climbing significantly accelerated the growth rate of BMD in rats in the growth phase [80]. In addition to weight-bearing ladder climbing, Fang et al. [81] discovered that the addition of an external load of 12% of bodyweight during moderate-intensity treadmill training significantly increased bone formation, improved the microstructure of trabecular bone, and preserved the structural and mechanical properties of cortical bone in female rats in the growth phase. However, when the additional weight was too high, it had no beneficial impacts on the multiscale properties of cortical bone or the microstructure of trabecular bone [81].

### 3.1.2 Effects of resistance training on the bone mass of middle-aged and elderly individuals

For premenopausal and postmenopausal women, a combination exercise regimen (impact training and resistance training) is the greatest option for maintaining and increasing BMD [82, 83]. Researchers have gradually discovered in recent years as the study population of elderly individuals has continued to grow that resistance training alone or in combination may be the best option for elderly individuals to prevent bone loss and even increase BMD in the lumbar spine and femoral neck [84]. Compared with endurance exercise, resistance exercise has higher requirements regarding the cardiopulmonary function and muscle strength of the individual being trained. Therefore, for weak elderly individuals and patients with other basic diseases or severe OP, combining resistance training with other types of exercise is a common exercise prescription mode. Furthermore, both low- and high-load training may increase BMD equally well when the training volume is comparable [85]. Therefore, for the aforementioned vulnerable groups, low-load, high-repetition resistance training might constitute another potential alternative exercise program. For elderly patients with OP, degeneration of sensory function and an increased risk of falling and fracture often appear together. Researchers have suggested that weight-bearing exercise, especially sustained resistance training, should be combined with aerobic exercise to improve balance, flexibility and coordination to improve BMD, enhance proprioception and body control ability, and reduce the risk of falling and the morbidity and mortality of fractures caused by falls [86]. In addition, in models of naturally aged female rats [87] and elderly female rats undergoing ovariectomy [88], anti-resistance training has also been confirmed to improve the BMD and bone microstructure of the rats.

### 3.1.3 Effects of resistance training on bone mass in special populations

Studies have confirmed that resistance training can increase lumbar spine and femur BMD in patients following kidney transplantation [89], patients with HIV infection [40] and patients with cancer [41]. Obesity and bone health have a complex relationship. Increased body weight has been shown to enhance bone mass, BMD, and BMC in numerous earlier research. However, metabolic and endocrine disorders caused by obesity inhibit osteogenic differentiation and induce OP and poor fracture healing. Weight-bearing ladder climbing can significantly reduce the bone loss caused by obesity and increase the trabecular bone BMD level in rats [90]. In addition, the increases in the BV/TV, Tb.N, and Tb.Th indices accompanied by decreases in Tb.Sp and SMI after resistance training also demonstrated that resistance training improved the bone microstructure [90]. In addition to the effects on those with obesity, Yamamoto [91] and Ikedo [92] observed similar positive effects of resistance training in diabetic rats that were induced in different manners. In short, we have found that either resistance training alone or in combination with other training modes can effectively recover the destruction of BMD and bone microstructure caused by a variety of pathological factors.

**Figure 3.**
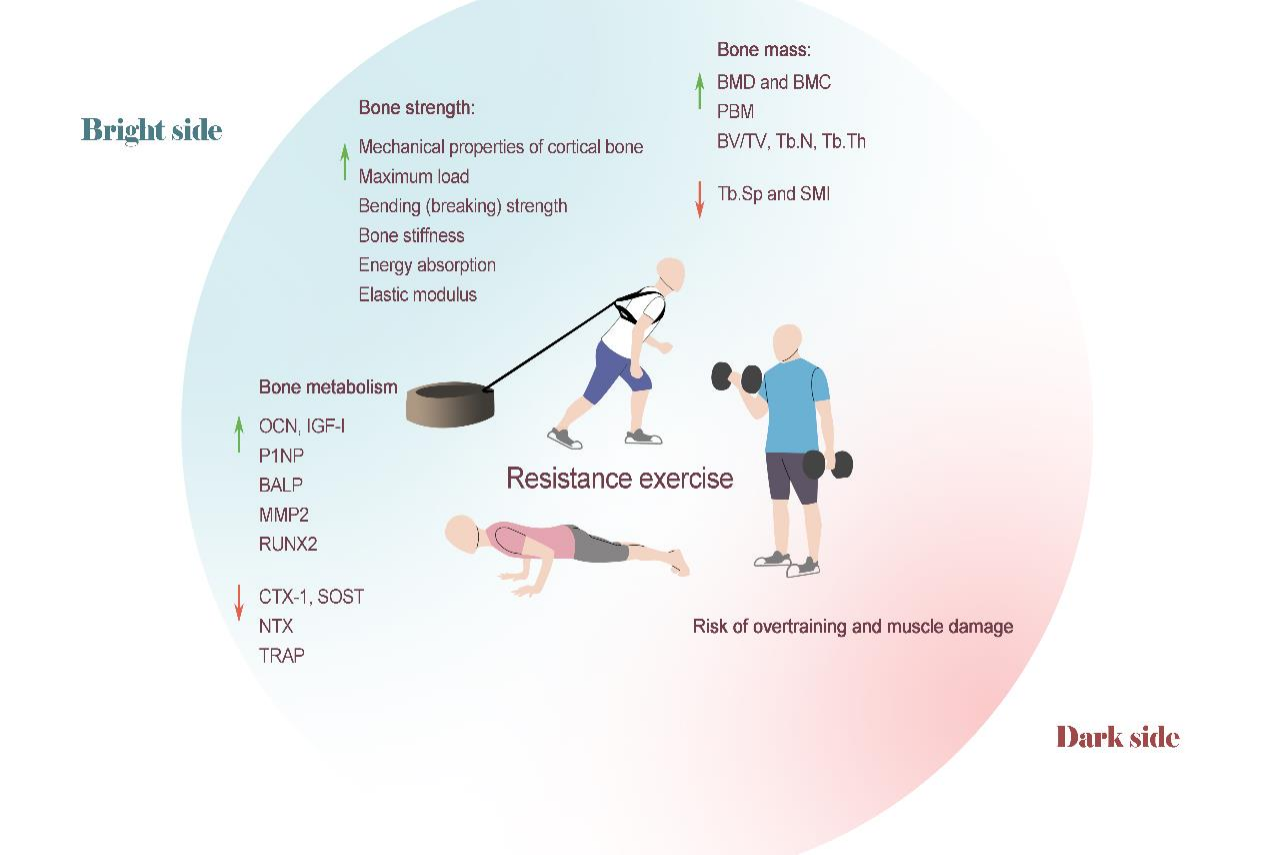
Resistance training on bone health. The improvement of BMD and bone microstructure from engaging in resistance training has been fully confirmed in various populations, and the effect has a significant correlation with the amount of training. Current studies support the positive effect of resistance training on bone strength in animal models and bone metabolism in humans. However, typical resistance training loads are difficult for first-time practitioners, placing them at risk of overtraining syndrome and muscle damage.

In summary, the improvement in BMD and bone microstructure caused by resistance training has been fully confirmed in various populations, and those effects are significantly correlated with the amount of training performed. However, the improvement effects on multiple-site local BMD have been inconsistent, which could be due to the specific method of resistance training that was utilized or the different populations. As mentioned above, under the premise that the total training volume is equated, both high-load and low-load resistance training can play a considerable role in bone health. Therefore, we encourage different populations to choose an appropriate resistance training program for their actual conditions to prevent or treat bone mass loss and bone microstructure damage caused by physiological or pathological factors. In animal studies, the resistance training load of rats could only be defined by the number of climbs or the amount of weight, and the training load is dispersed throughout the entire body. However, in actual applications, people mostly train with resistance exercises that target specific body parts (i.e., pure upper or lower limb exercises). Therefore, it is questionable to what extent rodent resistance training can mimic human resistance training (Fig. 3).

### 3.2 Effects of resistance training on bone strength

Resistance training causes changes in bone geometry that enhance its mechanical strength. Joo et al.[80] used three-point bending to test the biomechanical properties of the tibia in rats after 6 weeks of resistance training, and the results showed that the maximum load, bending (breaking) strength and bone stiffness indices were significantly improved, and bone mechanical strength was enhanced, without sex differences. Other studies have also reported improvements in bone biomechanical properties after weight-bearing exercise in various rat models, including neonatal diabetic rats [91], aged female rats [87] and ovariectomized postmenopausal rats [88, 93]. The benefit for bone strength was greater when resistance training was combined with other therapies. Tang et al.[94] combined myostatin polyclonal antibody injection with weight-bearing running in rats. Weight-bearing exercise alone resulted in a significant increase in the rats' elastic modulus and energy absorption at the end of the 8-week intervention, whereas injection of polyclonal antibodies resulted in a significant improvement in more biomechanical properties, such as maximum load, bone stiffness, elastic modulus, and energy absorption[94]. In conclusion, the current research results have supported the positive effect of resistance training on bone strength in rats, but likely because of the limitations of current experimental technology, there remains a dearth of relevant, high-quality human experimental evidence (Fig. 3).

### 3.3 Effects of resistance training on bone metabolism

### 3.3.1 Effects of resistance training on bone metabolism in middle-aged and elderly individuals

Hinton et al. [95, 96] reported that 12 months of resistance training promoted BMD increases in middle-aged men with low bone mass, and serum CTX concentrations were found to be significantly decreased at 6 months. At 12 months, OCN and insulin-like growth factor-1 (IGF-1) concentrations were increased significantly, and the concentration of SOST concentrations were decreased significantly [95, 96]. The changes in these indices indicate that a resistance training intervention has anti-absorption and anabolism promotion effects on the bones of middle-aged men. Resistance training is an important means of improving BMD in postmenopausal women with low bone mass. Pasqualini et al.[97] found in their clinical study that, after three months of weight-bearing and resistance training, PINP concentrations and the number of circulating osteoblasts in postmenopausal women were significantly increased, but CTX-1 concentrations were not significantly increased. In addition, weight-bearing exercise, alone or in combination with endurance exercise, could promote an increase in BALP in OP patients, reduce NTX concentrations, and maintain a positive balance of bone metabolism[98]. Exercise can effectively induce the production and secretion of muscle irisin. The secretion of irisin decreases gradually with aging of the body, and anti-resistance exercise training improves the secretion level to a certain extent [99]. Therefore, irisin could delay and prevent the loss of bone mass in middle-aged and elderly people.

In bone tissue, matrix metalloproteinases (MMPs) are an important coupling factor for bone remodeling, and they play an important role in the processes of osteoblast differentiation, osteoid dissolution, and osteoclast recruitment and migration [100]. Aging [101, 102] and ovariectomies (menopause) [93] both led to a decrease in MMP-2 activity in rats. Studies have shown that, after a certain period of resistance training, the activity of MMP-2 in the bone tissue of aged rats, ovariectomized rats and normal rats was significantly increased, and the positive balance of bone metabolism was promoted [93, 101, 102]. Dornelles's team found that the combination of resistance strength training and raloxifene (Ral) alleviated bone loss in ovariectomized rats [88] and naturally aging female rats[87] and improved the BMD and degeneration of bone microstructures. In addition, serum BTM (ALP, TRAP, OCN, SOST, etc.) concentrations and runt-related transcription factor 2 (Runx2) protein markers were positively affected. In 2020, the team also confirmed that strength training can promote the repair of fractures in aged perimenopausal rats by regulating the expression of genes related to bone remodeling and can improve the bone microstructures and physicochemical properties during the repair process [103].

### 3.3.2 Effects of resistance training on bone metabolism in special populations

Weight-bearing resistance exercise can improve bone loss and the negative balance of bone metabolism caused by severe hemophilia A [104] and spinal cord injury [105] and can improve the BTM indices of patients. Compared with long-term regular exercise, the acute effects of a single bout of resistance training on bone turnover and BTMs seem to be specifically related to factors such as sex and age. A single bout of resistance training was associated with reduced ALP concentrations in elderly individuals, while the combination of resistance and impact training had no effect on the OCN and BALP concentrations of middle-aged individuals, but it caused a significant decrease in CTX concentrations[106]. Therefore, there is no definitive conclusion about the acute effects of a single bout of resistance training on bone metabolism in all types of people, and a large number of additional studies are needed to support this type of exercise [106]. Notably, in the process of resistance training, an insufficient resistance load might not achieve the expected training effect, but when the load is too large, it can have a negative effect. Fu et al. [107] found that weight-bearing training with an appropriate load could promote the repair and regeneration of the necrotic femoral heads by regulating OPG/RANK/RANKL, while an excessive load adversely affected bone formation and remodeling, thereby inhibiting its recovery process.

In conclusion, resistance training positively regulates human bone metabolism by preventing bone resorption and boosting bone formation, as seen by alterations in BTMs concentrations in bodily fluids. Animal studies have further confirmed the positive promoting effect of resistance training on bone metabolism. However, unlike endurance exercise, resistance training can also regulate bone metabolism by regulating the activity of MMP (Fig. 3).

## 4 Speed and impact training and bone

Speed training refers to an exercise mode that quickly reaches maximal velocity in a short time, and it is also known as quick burst exercise. The 100-meter dash represents the qualities of a typical speed training exercise. Impact training is a form of exercise in which the contact points generate ground reaction forces on the body during movement, thus causing the bones and joints to bear transient repeated load stresses. For example, at the moment of landing on the ground or hitting a ball, the limbs will also bear the reaction forces from the outside. Therefore, jumping, football, basketball, tennis and other sports can be included in the scope of impact exercise. These two exercise modes have many similarities. From the perspective of energy metabolism, speed exercise and impact training are mostly anaerobic exercise modalities. Second, sprinting, football, basketball and other sports are known for their fast pace, fierce confrontation, and strong competitiveness. They require higher physical functioning of the participants and have a high probability of causing sports injuries. However, speed and impact training provide excellent mechanical stimulation to the bone; therefore, such sports are also considered effective bone-building exercises (Fig. 1).

### 4.1 Effects of speed and impact training on bone mass

#### 4.1.1 Effects of speed and impact training on bone mass in children and adolescents

Soccer, known for its intense pace and high competitiveness, is considered a type of sport that positively affects bone mass during growth. Lozano-Berges et al. [108] found that childhood soccer practice had a significant impact on BMC and BMD, particularly in the majority of weight-bearing regions, such as the lumbar spine, hip, and leg, where BMC and BMD were higher. Furthermore, even for non-weight-bearing bones, such as the radius, long-term studies have demonstrated that high-intensity impact training contributes more to the increase in BMD among adolescents than does low-intensity exercise [109]. Jumping has less of a load and is safer than competitive football. Repeated and multiple jump training causes the bones to bear the impact of body gravity and to exert a mechanical stimulation effect. Nine months of jump training improved bone geometry in young male athletes [110]. In addition, teens who participated in impact sports, such as football and basketball, had a significantly lower risk of stress fractures than those who participated in swimming [111]. Speed and impact sports are characterized by fierce competitiveness. Compared with other types of sports, their participants are at relatively high risk of acute sports injuries, such as bone fractures. Severe acute injury will inevitably cause permanent damage to the body and even directly affect the careers of professional athletes. As mentioned above, young athletes who have been engaged in endurance sports, such as swimming and cycling, for a long time face the threat of bone loss due to the lack of sufficient load stimulation on their bones. In response to this fact, Vlachopoulos et al. [112] illustrated that jump training for nine months improved BMD, bone hardness, and muscle health in male adolescents who regularly participated in swimming and cycling significantly reducing the risk of stress fractures.

Animal studies have confirmed that the response of bones to mechanical stimulation is different at different stages of mouse growth and that the sensitivity of bones to mechanical loading in young mice is significantly higher than that in adults and in older mice [113]. It was reported in early studies that jump training can significantly promote the growth of the BMD and BMC of femurs in 4-week-old mice during the growth phase [114]. Mustafy et al. [115] tested the effect of exercise under different loads on bone mass in growing mice. The results showed that, after 8 weeks of intervention, including walking (low load), uphill running (medium load), and jumping (high load), the impact load in adolescence partially reduced the bone growth rate but increased the BMD and improved bone mass and biomechanical properties, and the effect in the jumping training group was the most significant [115]. Therefore, impact training with a higher load seems to be more beneficial to bone growth and development.

#### 4.1.2 Effects of speed and impact training on bone mass in middle-aged and elderly individuals

Studies have shown that impact training and resistance training are effective methods to stimulate the bones of adults, especially high-intensity impact training, which has the greatest osteogenic potential for most adults and seems to be the best exercise modality to enhance bone mass [86]. Jumping is the most common and simplest impact exercise. Compared with competitive sports, it is gentler and safer and is more suitable for the general public, especially elderly individuals. Bolam et al. [116] reported the protective effect of jump training 4 times per week for 9 months on hip and greater trochanter BMD in elderly men, and stated that it could prevent bone loss caused by aging and that the training effect is dose-dependen. However, in that experiment, even the relatively intense jump training only delayed the rate of decline in BMD in elderly men and did not improve BMD. The effects of jump training might differ greatly between sexes. In another study, 16 weeks of jump training effectively improved body function, BMD and bone microarchitecture in multiple body sites in postmenopausal women [117]. To explore the effects of high intensity and high load exercise training on the bones of elderly individuals, Suominen et al. [118] recruited a group of middle-aged and elderly sprinters who were able to complete strenuous exercise, and they conducted 20 weeks of high-intensity combined strength and sprint training. In that study, the BMD, BMC, cortical thickness (Ct.Th), cross-sectional area, and bone biomechanical properties of the tibia of middle-aged and elderly athletes receiving high-intensity combined training were significantly improved [118]. This result suggests that adaptive changes in bone and improvement in bone mass are possible with sufficient external exercise loads, although an age-related decline in bone health is inevitable.

In animal experiments, researchers have found that jump training can improve the BMD and BMC of ovariectomized female Wistar rats and has a positive effect on BV/TV, Tb.Sp and Tb.N and other indicators, thereby effectively preventing bone loss and bone microstructure damage after oophorectomy [119, 120]. In addition to jump training, free-fall impact training is also a type of impact movement training that can be achieved in animal models. It has been reported that free-fall impact training can increase the BMD of rats and improve their bone microstructure [121]. However, the different free-fall impact time intervals appeared to cause slightly different results: compared with the control group, the free-fall group with an interval of 10 seconds experienced a significant reduction in Tb.Sp, while the free-fall group with an interval of 20 seconds experienced a significant reduction in Tb.N [121].

#### 4.1.3 Effects of speed and impact training on bone mass in special populations

According to reports in the literature, the femoral and whole-body BMD levels football players after long-term and systematic training are 7.3% to 37.4% higher than those of the general population of the same sex and corresponding age group [122]. Even more surprising is that, despite an age difference of nearly 50 years, the BMD of the trochanter and legs of a lifetime-trained older soccer player was higher than that of an untrained 18- to 30-year-old individual [122]. Prostate cancer is a common malignant tumor in elderly men, and androgen deprivation therapy is a conventional and effective treatment. However, the lack of androgen often leads to bone loss and a rapid decline in muscle strength. Uth et al. [123] followed up and evaluated the effects of 5 years of long-term football training on the physical condition of elderly men with prostate cancer undergoing androgen deprivation therapy. The results showed that, over time, the bone mass, muscle strength, endurance, and other physical characteristics of all the subjects inevitably deteriorated. However, compared with the control group, the BMD level of the right femoral neck of those who regularly participated in football was significantly better, indicating that football could at least delay the bone loss of these patients. Myocardial infarction is an important factor inducing OP fracture. Based on this information, Kanazawa et al. [124] confirmed that sprinting as a form of speed training could delay the decrease in BMD caused by myocardial infarction in apolipoprotein E-deficient mice.

**Figure 4.**
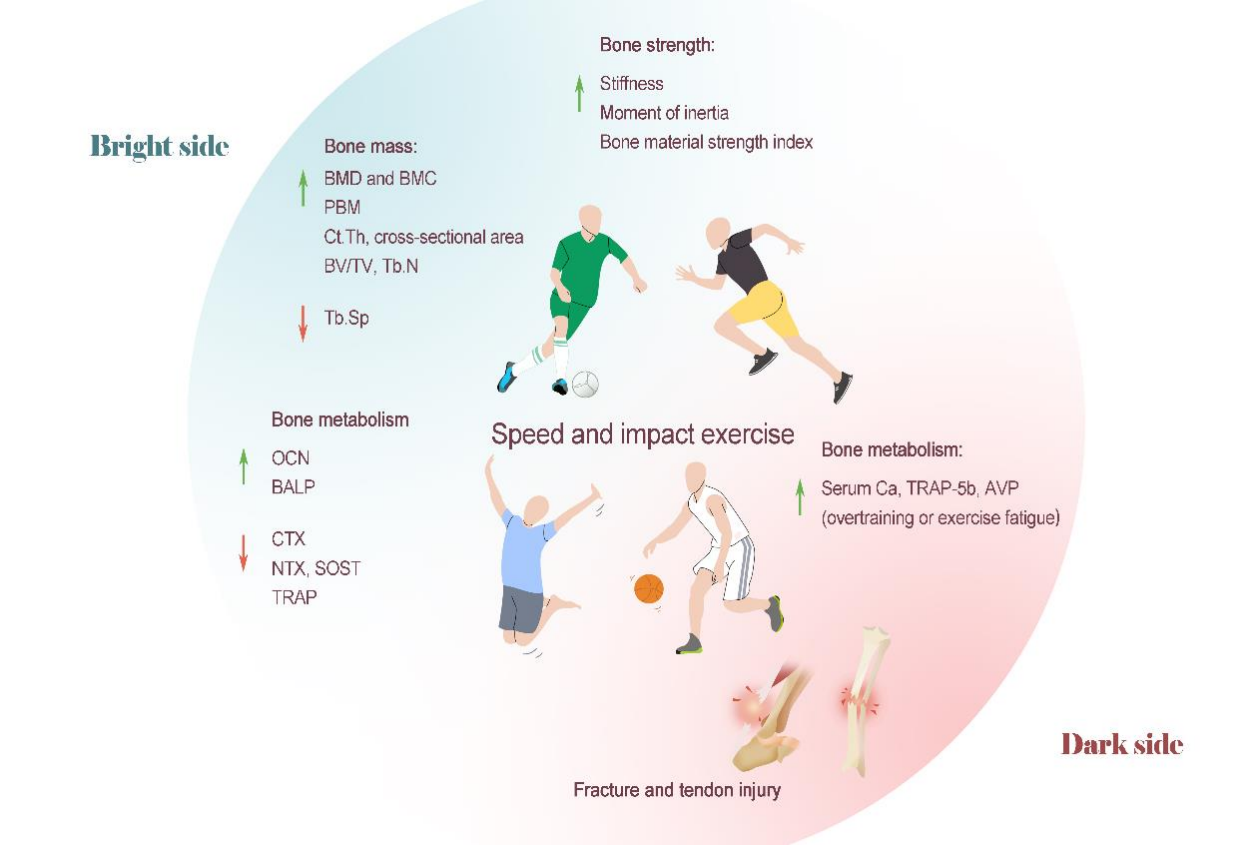
Speed and impact training on bone health. High-intensity impact training seems to be the most effective way to promote the bone growth and development of teenagers, improve the bone mass of middle-aged and elderly individuals and OP patients and increase BMD and BMC. Speed and impact training can also improve bone biomechanical properties. The impact training program has excellent effects on promoting bone formation and preventing bone loss. However, speed and impact training are accompanied by intense aggression and a significant increase in the risks of various acute and chronic sports injuries.

In summary, high-intensity and severe impact training seems to be the most effective way to promote the bone growth and development of teenagers, improve the bone mass of middle-aged and elderly people and OP patients and to increase BMD and BMC. The combination of impact and resistance training will often achieve better results than either training modality in isolation. Therefore, for the general population, impact and resistance training should be recommended to prevent or improve loss of bone mass. However, for the weak and elderly individuals, vigorous sports, such as sprinting and ball games, are no longer suitable. The relatively safe jump training is a more popular sports training method. In addition, middle-aged and elderly individuals should be encouraged to combine jumping movements with resistance training to delay the decline in age-related body function to the greatest extent and to improve their musculoskeletal condition. Currently, for people of different ages, sexes and physical health conditions, there could be differences in the effect of impact training. Although in-depth animal studies have been conducted, more mechanisms are needed to determine the appropriate training load and frequency for different populations (Fig. 4).

### 4.2 Effects of speed and impact training on bone strength

In animal studies, jump training can improve the femoral stiffness [119] and moment of inertia [120] of ovariectomized rats. The bone material strength index (BMSi), which is different from three-point bending, is noninvasive and can be used for human research. Sundh et al. [125] confirmed that 3 months of high-intensity unilateral jump training could significantly increase the tibial BMSi value in postmenopausal women, but there was no significant change in the parameters of BMD and bone microstructure in the tested population. Therefore, the authors proposed that this might be a new mechanism that rapidly increases the mechanical strength of bone under the condition of increased weight bearing exercise which occurs prior to changes in BMD and bone microstructures [125] (Fig. 4).

### 4.3 Effects of speed and impact training on bone metabolism

#### 4.3.1 Effects of speed and impact training on bone metabolism in children and adolescents

Speed and impact training regulate the process of bone remodeling in various populations. First, jump training significantly increased the serum BALP concentrations of teenagers and children [126]. According to the "2020 WHO Guidelines for Physical Activity in Children and Adolescents (5-17 years old)", physical activity is highly important for adolescent bone health; therefore, the recommendations state that children and adolescents should engage in at least 60 minutes of vigorous physical activity (mainly aerobic activity) each day on average, as well as vigorous physical activity and musculoskeletal resistance training at least three days per week [127].

#### 4.3.2 Effects of speed and impact training on bone metabolism in middle-aged and elderly individuals

Vasto et al. [128] designed a new trampoline exercise called SuperJump. Their experimental data showed that 20 weeks of SuperJump activity significantly increased serum OCN concentrations in healthy women while significantly reducing CTX concentrations [128]. On this basis, they further pointed out that the increased secretion of endogenous intestinal glucagon-like-peptide-1 (GLP-1) and glucose-dependent insulinotropic polypeptide (GIP) after jumping exercise was involved in the mechanism of improving bone health and maintaining glucose metabolism homeostasis in postmenopausal women [129]. In fact, regular jumping exercise could also significantly increase serum OCN concentrations and can improve bone metabolism in postmenopausal women [130]. With increasing age, the decreased sensitivity of aging bones to mechanical load stimulation and the damage to their mechanical stimulation adaptive ability might be important related factors that cause OP in elderly individuals. Recently, some scholars have noted that a high-impact exercise program can restore the mechanical adaptive ability of bones in elderly individuals and affect osteogenic-related signals, which could be beneficial to the bone health of elderly individuals to a certain extent [131].

Okubo et al. [119] reported that 12 weeks of jump training could cause an increase in serum OCN concentrations in ovariectomized rats. Aveline et al. [120] found that jump training alone increased the serum OCN concentrations of ovariectomized rats and reduced the apoptosis of bone cells. In addition to the effects mentioned above, the combined intervention of jump training and strontium ranelate intake also affected the concentrations of ALP and NTX [120]. However, compared with jumping exercise, free-fall impact exercise with higher impact forces was also found to increase OCN concentrations, decrease NTX concentrations, and inhibit the expression of SOST mRNA in bone tissue in adult male Wistar rats [121]. It was further proven that the positive promoting effect of impact training on bone metabolism might have a positive correlation with the intensity of the impact loads.

#### 4.3.3 Effects of speed and impact training on bone metabolism in special populations

Although speed and impact training have shown excellent effects in promoting osteogenic metabolism, in fact, they can also easily cause fatigue for participants due to the intense and vigorous nature of the exercises. Senda et al. [132] conducted a single 5-h continuous mixed high-intensity exercise session for well-trained soldiers and detected that their blood calcium, serum TRAP-5b and arginine vasopressin (AVP) concentrations significantly increased, accompanied by a significant decrease in NTX-1 concentrations. This finding suggests the enhancement of bone resorption metabolism. High-intensity running, which is representative of a speed exercise, can inhibit the activity of TRAP and increase the activity of ALP in OP mice secondary to myocardial infarction, thus improving the femoral BMD status of mice after myocardial infarction [124].

In summary, an impact training program has excellent effects on promoting bone formation and preventing bone loss. It is of great significance to encourage young children in their growth and development period to participate in all types of intense sports to promote bone health. For middle-aged and elderly people, gentle and safe jump training seems to be the best choice for physical exercise to inhibit bone resorption, promote the positive development of bone metabolism, and prevent or treat OP (Fig. 4).

## 5. Flexibility and balance training and bone

Flexibility refers to the range of motion of human joints and the elasticity and extension ability of ligaments, tendons, muscles, skin and other tissues. Balance is a person's ability to maintain stability or the ability to maintain the center of gravity on the support surface. Flexibility and balance are integral parts of overall physical quality, and both can be enhanced through systematic training. Exercise training aimed at strengthening the flexibility and balance of the body is called flexibility and balance training. Flexibility and balance training mostly rely on the aerobic energy system, similar to aerobics, yoga, Pilates, and Chinese traditional martial arts (Tai Chi, Baduanjin, etc.), which have also been considered good exercise modalities to improve soft tissue flexibility, body balance and coordination, and those modalities have spread around the world (Fig. 1).

### 5.1 Effects of flexibility and balance training on bone mass

#### 5.1.1 Effects of flexibility and balance training on bone mass in children and adolescents

Flexibility and balance training mainly stimulate soft tissues, such as skeletal muscle and fascia, and are not typical methods for bone-building exercise. However, studies in recent years have found that flexibility and balance training can also promote bone health. Rhythmic gymnastics have been shown to improve bone health in teenagers and children. Jurimae [133] and Tamoliene [134] both reported that young athletes performing professional rhythmic gymnastics showed higher BMD and BMC levels and can overcame the negative effects of negative energy balance and low body fat mass due to excessive activity levels.

#### 5.1.2 Effects of flexibility and balance training on bone mass in middle-aged and elderly individuals

Compared with artistic gymnastics requiring strong skills and techniques, lower threshold yoga and Pilates have more extensive popularity among the general public. It has been confirmed in previous studies that yoga and Pilates can improve the flexibility and balance of the body. However, there have been no clear conclusions about the effects of these two factors on BMD and BMC. Most relevant studies have focused on postmenopausal women, and the reported results have been inconsistent. Some studies have reported that years of yoga practice can prevent bone loss in elderly individuals (mainly women) and to some extent improve bone quality and BMD [135]. Although factors such as small sample sizes and insufficient intervention times might exist, no significant improvement in the BMD of subjects after yoga or Pilates training has been observed in most of the existing studies. Pilates and yoga have not been effective at improving BMD in adult women, as concluded by a meta-analysis [136]. Tai Chi, Baduanjin, Wuqinxi and other traditional Chinese martial arts have a history of thousands of years, and their health-care effects of building up the body and prolonging life have been widely recognized. In recent years, the bone-strengthening function of the traditional Chinese exercise method represented by Tai Chi has gradually attracted extensive attention from experts and scholars. Tai Chi has been shown to effectively improve bone health in elderly, perimenopausal, and post-menopausal women and to significantly increase BMD in the lumbar and proximal femoral regions in this population [137-139]. In addition to Tai Chi, Baduanjin has also been shown to improve BMD in postmenopausal women, thus showing great potential for preventing and treating postmenopausal OP in middle-aged and elderly women [140].

**Figure 5.**
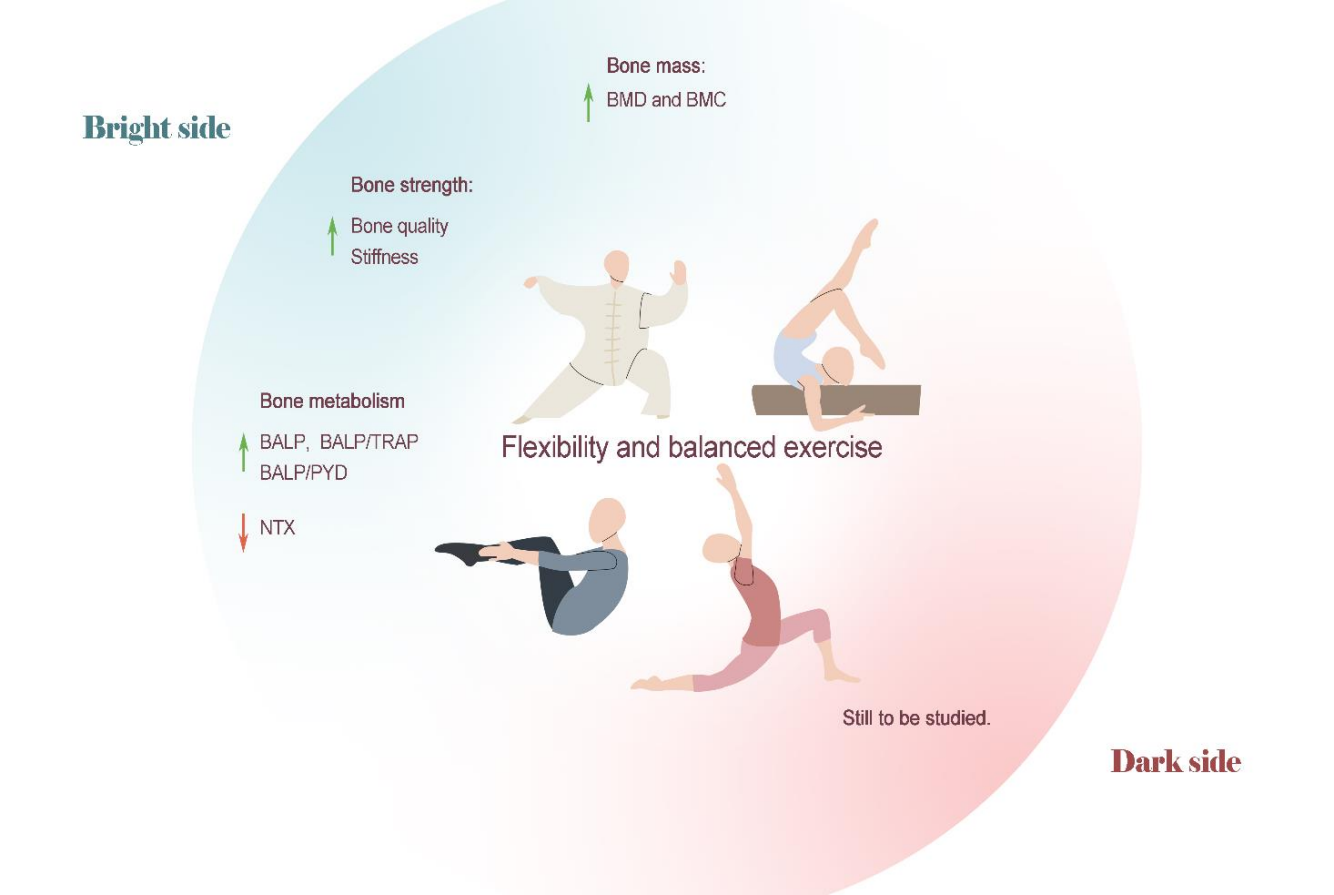
Flexibility and balance training on bone health. Rhythmic gymnastics can promote bone growth and increase BMD, BMC and PBM in children and adolescents. Chinese traditional martial arts, represented by Tai Chi, can effectively reduce the BMD decrease caused by various factors. Tai Chi has benefited osteogenic metabolism and positively influenced bone remodeling in a variety of population studies. Gymnastics can significantly improve bone quality and stiffness index.

In summary, rhythmic gymnastics training can promote bone growth and increase BMD, BMC and PBM in children and adolescents. Whether yoga and Pilates improve BMD is not entirely certain. However, this type of exercise undeniably has advantages over other types of exercise in increasing the flexibility of soft tissues, improving balance and coordination, and enhancing proprioception. For elderly individuals, OP patients and other high-risk groups, this type of exercise can reduce the risk of falls and the incidence and mortality of fractures. Chinese traditional martial arts, represented by Tai Chi, can effectively reduce the BMD decline caused by various factors and are a cost-effective and effective OP prevention measure (Fig. 5).

### 5.2 Effects of flexibility and balance training on bone strength

In young people, medication is another important OP-inducing factor, in addition to physiological and pathological factors. Among medications, opioids (such as morphine) are strong, addictive drugs that are difficult to quit once taken, and long-term intake of opioids greatly damages bone health and induces severe OP [141]. Ding et al. [142] attempted to use gymnastics training to prevent and treat opioid-dependent OP in women. Fortunately, after eight months of gymnastics training, the bone quality and stiffness index of the exercise group were significantly increased, demonstrating that gymnastics could effectively prevent and treat drug-induced OP[142] (Fig. 5).

### 5.3 Effects of flexibility and balance training on bone metabolism

#### 5.3.1 Effects of flexibility and balance training on bone metabolism in middle-aged and elderly individuals

Gandolfi et al. [143] found that, although 20 weeks of Pilates improved the quality of life of older women, their BALP and CTX-1 concentrations were not changed [143]. Tai Chi, however, had a positive effect on serum BTMs concentrations in postmenopausal older women: BALP concentrations were increased, and BALP/TRAP ratios were improved[144]. Additionally, Shen et al. [145] found that the serum BALP/PYD ratios of the 24-week Tai Chi group were also significantly higher than those in the resistance training group and that they were greater than those at baseline. Although the relatively low adherence of the elderly in the resistance training group in this trial might have affected the experimental results, these results are sufficient evidence that Tai Chi exercise improves osteogenic metabolism in elderly individuals.

#### 5.3.2 Effects of flexibility and balance training on bone metabolism in special populations

Bone loss often occurs in patients with breast cancer. In particular, for breast cancer survivors after hormone therapy, the rate of bone loss is greatly increased, making it extremely easy for OP to occur. The study found that, after 12 weeks of Tai Chi training, serum BALP concentrations of breast cancer survivors increased and NTX concentrations significantly decreased; that is, Tai Chi alleviated bone mass loss in breast cancer survivors by increasing bone formation and reducing bone resorption [146].

Although there have been few studies on the relationships between flexibility and balance training and bone metabolism, we found that the effects of such exercise on bone metabolism are consistent with those on BMD mentioned above. Tai Chi has positively influenced bone remodeling in a variety of population studies. Different from the other three types of exercise, under the current conditions, flexibility and balance training requires a higher skill level and is more complex, and thus, it is relatively difficult to carry out animal research. Therefore, the relevant mechanisms need further research (Fig. 5).

**Table 2:** Effects of different types of exercises on bone structure and function.

Exercise Type	Bone Mass	Bone Strength	Bone Metabolism
Endurance exercise	Does not exert positive effects	No significant improvement	Promotes the positive balance of bone metabolism in middle-aged and elderly individuals, but has no positive effect on adolescents and children
Resistance training	Improves BMD and BMC in various populations	Improves the biomechanical properties of bone	Prevents bone resorption and boosts bone formation in various populations
Speed and impact training	Improves BMD and BMC in various populations	Improves the biomechanical properties of bone	Has excellent effects on promoting bone formation and preventing bone loss
Flexibility and balance training	Promotes bone growth and maintains BMD and BMC in children, middle-aged and elderly individuals	Improves bone strength in drug users	Has a positive influence on bone remodeling; further research is needed

## 6. Redox reactions mediate motility and regulate bone homeostasis

### 6.1 Introduction to oxidative stress

Throughout the lifespan of the human body, oxygen is used for aerobic respiration at all times to supply energy for all of life’s activities. In this process, a series of redox reactions occurs. In addition to releasing energy, these reactions also produce a large number of oxidation byproducts, namely biological free radicals, also known as "oxidants". There are two main groups of free radicals in living organisms: reactive oxygen species (ROS) and reactive nitrogen species (RNS). The former includes superoxide anions (O_2_ •^-^), hydroxyl radicals (•OH), and others. The latter includes nitric oxide (NO), nitrogen dioxide (NO2) and others. ROS and RNS contain free radicals, and their reaction derivatives, such as hydrogen peroxide (H_2_ O_2_ ) and peroxynitrite (ONOO^-^), also belong to the above category. In fact, all aerobic cells in an organism produce ROS and RNS. ROS are usually produced in the cytoplasm, mitochondria, endoplasmic reticulum and lysosomes, while RNS are mainly the result of amino acid metabolism [147]. The accumulation of free radicals damages human tissues and cell structures, resulting in protein and lipid peroxidation and DNA damage. To combat these adverse effects, the body has developed corresponding antioxidant systems. There are two types of antioxidant systems in cells. One is the enzymatic antioxidant system, including superoxide dismutase (SOD), catalase (CAT), glutathione peroxidase (GSH-PX), and others. The other nonenzymatic antioxidant system includes vitamin C, vitamin E, glutathione (GSH), and uric acid. Under normal circumstances, the peroxides produced in living organisms can be almost completely removed by the antioxidant systems to achieve homeostasis. However, when an imbalance between oxidation and antioxidation occurs in the body, peroxide cannot be removed in time, resulting in a state of oxidative stress. Jones [148] defined oxidative stress as "an imbalance between oxidants and antioxidants that favors oxidants, leading to disruption of redox signaling and control, and/or molecular damage". Since the first discovery of free radicals in organisms in 1954, a new area of study has opened and has inspired researchers to explore the life sciences [149]. Since then, researchers have conducted more in-depth and extensive research in this field. Today, a large number of studies have confirmed that the formation of free radicals and oxidative stress injury play an important role in the process of biological aging[150, 151]. In addition, oxidants are closely related to the occurrence of a variety of age-related noncommunicable diseases, such as cancer, diabetes, cardiovascular disease and neurodegenerative diseases [152, 153].

### 6.2 Oxidative stress and bone homeostasis

**Figure 6.**
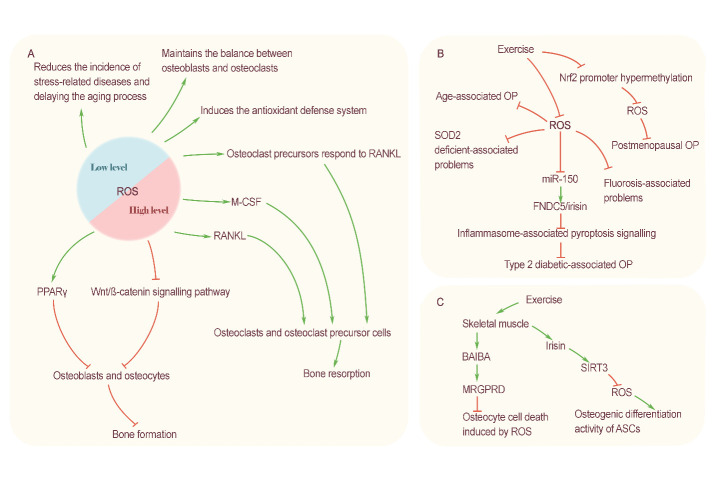
Oxidative stress mediates exercise to regulate bone homeostasis. A) Low concentrations of ROS act as signaling molecules to maintain bone homeostasis and the balance between osteoblasts and osteoclasts, promote stress resistance processes, such as the induction of the antioxidant defense system, reduce the incidence of stress-related diseases and delay the aging process. High concentrations of ROS play an important role in the pathological changes during the progression of OP. Excessive ROS production can enhance the responsiveness of osteoclast precursors to RANKL, stimulating M-CSF and RANKL expression and increasing the RANKL/OPG ratio. Activation of the differentiation of osteoclast precursor cells increases the number and activity of osteoclasts and enhances the activity of bone resorption. An increase in ROS production inhibits the expression of the Wnt/β-catenin signaling pathway but activates the expression of PPARγ and supports adipogenesis at the expense of osteoblastic genesis. Inhibiting the differentiation of osteoblasts and inducing the apoptosis of osteoblasts and osteocytes inhibits bone formation. B) Moderate regular exercise can improve the body's antioxidant defense ability and inhibit excessive ROS. Exercise has a prevention and treatment effect on OP caused by many factors, including age-associated OP, postmenopausal OP, and type 2 diabetes-associated OP. In addition, among bone health problems caused by some special factors, such as fluorosis and SOD2 deficiency, exercise has also been confirmed to play a good therapeutic role and protect bone from oxidative stress damage. C) Exercise-induced muscle factors regulate the link between oxidative stress and bone homeostasis. Irisin and BAIBA are secreted by skeletal muscle during exercise. Irisin upregulates SIRT3 expression, reduces excessive oxidative stress and improves mitochondrial autophagy in ASCs thereby improving the osteogenic differentiation activity of ASCs.BAIBA signals through MRGPRD, to inhibit mitochondrial breakdown in osteocytes, thereby protecting osteocytes from ROS-induced apoptosis.

In the skeletal system, OP caused by a negative balance in bone metabolism is typically representative of age-related degenerative diseases. As mentioned earlier, in addition to physiological aging, estrogen deficiency is also an important factor in inducing OP. Studies have shown that aging and estrogen level changes will disrupt redox balance and lead to oxidative stress. Therefore, many OP patients show a high level of oxidative stress, which plays an important role in the destruction of bone microstructures, bone loss and other pathological changes during the progression of OP [154, 155]. In fact, the results of a propensity score matching study also confirmed that oxidative stress status has a clear, positive correlation with the prognosis of hip fracture in the elderly [156]. Manolagas [157] proposed changing the mechanism of OP from "estrogen" to "natural aging and oxidative stress" and stated that excessive accumulation of ROS and oxidative stress are the basic mechanisms of OP occurrence and development. In the body, estrogen plays a crucial role as an antioxidant. Estrogen can, in fact, counteract the prosurvival effects of RANKL receptor activators on osteoclasts and ROS-induced osteoblast death in bone tissue [157]. Therefore, due to the decrease in estrogen levels after menopause, the damage to antioxidant capacity in the body can also explain the higher incidence of OP in postmenopausal women. Studies have shown that ROS can influence the physiological functions of osteoblasts and osteoclasts, which in turn can change the skeletal environment. First, oxidative stress can promote osteoclast precursor cell development, boost osteoclast activity and numbers, and then enhance bone resorption activity [158, 159]. Mechanistically, ROS enhance the responsiveness of osteoclast precursors to RANKL [160], stimulating macrophage colony-stimulating factor (M-CSF) and RANKL expression and increasing the RANKL/OPG ratio [161]. In addition, ROS can inhibit the differentiation of osteoblasts and induce apoptosis of osteoblasts and osteocytes [159, 162]. Studies have found that the increase in ROS inhibits the expression of the Wnt/β-catenin signaling pathway but activates the expression of peroxisome proliferator-activated receptor γ (PPARγ), which is a key molecule in adipogenesis and supports adipogenesis at the expense of osteoblastic genesis [157]. Unfortunately, compared with osteoclasts, the effects and molecular control of oxidative stress on osteoblast differentiation and function are not well understood. In conclusion, low levels of ROS act as signaling molecules to maintain bone homeostasis and the balance between osteoblasts and osteoclasts [159]. However, excessive ROS-induced oxidative stress damage also plays an indispensable role in bone metabolic imbalance and bone degenerative changes caused by various factors (Fig. 6).

### 6.3 Oxidative stress mediates exercise to regulate bone homeostasis

The role of regular and appropriate exercise in promoting physical health has been fully confirmed. However, the relationship between exercise and oxidative stress has been a popular and interesting issue for many years. A large number of previous studies have confirmed that sports can lead to an increase in ROS and oxidative stress. Prolonged endurance exercise, resistance exercise, high-intensity anaerobic exercise, or a single bout of aerobic exercise can lead to an inflammatory response and oxidative stress, which is suspected to be caused by an increase in proinflammatory factors and oxidative biomarkers in the skeletal muscle and blood [163, 164]. In turn, it can lead to cell damage, which is manifested as lipid peroxidation, amino acid carbonylation, and DNA damage. The main source of ROS during exercise is skeletal muscle, and the buildup of ROS is a key cause of exercise-induced exhaustion [165, 166]. However, at the same time, regular exercise training can cause significant adaptation of the body, which can improve the antioxidant defense system by upregulating the expression of SOD and GSH-Px genes and improving antioxidant capacity [163, 166]. In addition, it has been shown that exercise-induced oxidative stress plays a role in inducing cellular defense mechanisms, thereby reducing the incidence of stress-related diseases and delaying the aging process [167]. Some scholars have proposed that the relationship between exercise training and free radical substances can be explained by hormesis theory. Hormesis is a dose-response phenomenon characterized by low-dose stimulation and high-dose inhibition, resulting in a J-shaped or inverted U-shaped dose-response relationship [168]. In other words, low doses of exercise-induced ROS could promote stress resistance processes, such as the induction of the antioxidant defense system, which ultimately has a health-promoting effect, but with the onset of overtraining syndrome, the antioxidant system of the organism is maladapted and the redox balance of the body is damaged [168]. From this point of view, the consequences of oxidative stress caused by exercise are still a very controversial topic and are closely related to many factors, such as exercise load, exercise type, and the individual’s fitness level.

Sierra-ramirez et al. [169] demonstrated that moderate aerobic exercise reduced oxidative stress markers in aging BALB/c mice and significantly improved bone quality during aging. Studies have shown that oxidative stress is an important mechanism in the pathogenesis of T2DM and secondary OP. Skeletal muscle is the primary effector organ of exercise, and myocytokines produced during exercise have been confirmed to mediate the beneficial effects of exercise training through endocrine communication between skeletal muscle and a variety of tissues and organs, including bone [170]. Behera et al. [171] found that 8 weeks of aerobic running or exogenous irisin supplementation promoted bone formation in T2DM mice and improved trabecular microstructure and bone mechanical properties [171]. It was further confirmed that aerobic exercise induces skeletal irisin and plays a positive role in regulating the miR-150-FNDC5/pyroptosis axis by inhibiting oxidative stress injury [171]. Under diabetic conditions, exposure to advanced glycation end products (AGEs) retards growth of adipose-derived stem cells (ASCs) and inhibits the osteogenic differentiation of ASCs in a time - and dose-dependent manner. In a study conducted by Li [172] and colleagues, irisin treatment significantly suppressed intracellular ROS concentrations by regulating the oxidative stress state and mitochondrial function of ASCs under diabetic conditions, thereby improving the osteogenic differentiation activity of ASCs. In their study, it was demonstrated that irisin can reduce excessive oxidative stress in cells and improve mitochondrial autophagy, and sirtuin 3 (SIRT3) may be the key target molecule for irisin to play a role [172]. Similar to irisin, β-aminoisobutyric acid (BAIBA), another exercise-induced muscle factor, has also been shown to have promising osteoprotective characteristics. Studies have shown that BAIBA signals through its receptor, Mas-related G protein-coupled receptor type D (MRGPRD), to inhibit mitochondrial breakdown in osteocytes, thereby protecting osteocytes from ROS-induced apoptosis [173]. In addition, among bone health problems caused by some special factors, such as fluorosis [73] and manganese superoxide dismutase (SOD2) deficiency [174], aerobic exercise has also been confirmed to play a therapeutic role and to protect bone from oxidative stress damage. Recent studies have shown that epigenetic changes are closely related to the pathogenesis of OP. Chen et al. [44] reported that Nrf2 expression in femora was significantly inhibited in OP patients and ovariectomized mice, accompanied by increased DNA methyltransferase (DNMT)-1/DNMT-3a/DNMT-3b, Nrf2 promoter hypermethylation, and excessive accumulation of reactive oxygen species. Regular running exercise can effectively promote the demethylation of the Nrf2 promoter in ovariectomized mice, reactivate the antioxidant enzyme system, and prevent the loss of femoral BMD in a NrF2-dependent manner. In addition to animal studies, similar results were reported in a clinical study involving 171 women with postmenopausal bone loss. Qian et al. [175] found that 6 months of Tai Chi training combined with green tea polyphenol supplementation significantly reduced the concentrations of oxidative stress markers in the serum and urine of postmenopausal women with osteopenia. Since oxidative stress is an important mechanism of postmenopausal OP, Tai Chi exercise is expected to become a new means to improve bone health. In conclusion, moderate and regular exercise can enhance the antioxidant capacity of bone and inhibit the adverse consequences caused by oxidative stress (Fig. 6).

Based on the role of redox reactions in bone metabolism, it is clear that inhibiting oxidative stress or enhancing the antioxidant capacity of the body will be an important means to prevent and treat diseases and promote bone health. In addition to lifestyle interventions such as appropriate increases in exercise, maintaining a good sleep and rest schedule, and maintaining a nutritionally balanced diet, antioxidant supplementation has also been shown to provide significant benefits. Natural foods rich in antioxidant activity are promising sources of dietary antioxidants. The results of multiple clinical studies have confirmed that vitamin C, vitamin E, and soy isoflavones have excellent antioxidant effects and are beneficial to bone metabolism [155]. Data from the current study showed that supplementation with more than 500 mg of vitamin C and 50 mg of isoflavones per day was effective in improving BMD [155]. At the same time, the researchers also indicated that the synergistic effect of antioxidants when ingested in the form of whole antioxidant plant foods compared with the isolated antioxidant components alone, so the direct consumption of whole plant foods rich in antioxidants seems to have a better effect. For example, daily intake of prunes (>50 g) and/or their polyphenol extracts significantly improved bone health in postmenopausal women by reducing the secretion of malondialdehyde (MDA) and nitric oxide (NO), increasing the expression of antioxidant enzymes, inhibiting oxidative stress and the inflammatory response [176]. In addition, natural plant-derived substances such as resveratrol [177], arctiin [178], crocin [179], and pseurotin A [180] from Aspergillus fumigatus have been found to inhibit ROS and regulate oxidative stress in preclinical studies. In turn, those antioxidant effects have a positive effect on bone metabolism. In conclusion, antioxidants have broad application prospects for the prevention or improvement of age-related and postmenopausal OP. However, thus far, there are no relevant studies on alleviating bone oxidative stress caused by excessive exercise. In the future, the development of sports nutrition supplements or drugs will be a meaningful research direction.

In summary, a large number of research conclusions and clinical results have confirmed the positive effects of appropriate physical exercise on bone health. One of the important mechanisms of its effects is enhancement of the oxidative defense ability of bone tissue and inhibition of oxidative stress. Based on the dual effects of exercise on the oxidative stress response, the role of oxidative stress in exercise-mediated changes in bone metabolism has been gradually revealed. However, there is a lack of high-quality related research results, and the mechanism by which exercise induces/inhibits oxidative stress in bone tissue is still unclear. In addition, more clinical research on this aspect is also a direction for the future.

## 7. Conclusions

In summary, we have found that different types of exercise have different effects on bone due to their unique technical characteristics. Based on the current research, endurance exercise seems to have a limited promotion effect on the bone growth and development of teenagers and children. Although endurance exercise plays a certain role in preventing bone loss, it is not the perfect bone-building exercise. Resistance training and speed and impact training have been fully verified to be effective in promoting bone growth, maintaining bone mass and improving bone quality, and they are beneficial to the bone health of all types of individuals. Research data on flexibility and balance training are currently limited, but rhythmic gymnastics and Tai Chi have bright prospects for promoting bone health in multiple populations.

With the development of medicine, the therapeutic effects of exercise have been gradually recognized. Therefore, kinesitherapy is also being increasingly used as a common component within the comprehensive clinical treatment of a variety of diseases. Exercise prescriptions have also been developed. Exercise prescription is a personalized method to guide people to exercise in a purposeful, planned and scientific fashion. Evidence suggests that, in some cases, exercise therapy can be as effective as or even better than clinical treatment. A reasonable exercise prescription should include the modulation of many factors, such as frequency, intensity, time, and type, which should be selected scientifically based on an understanding of the characteristics of various types of exercise and the actual situation of the individual. Therefore, different groups should follow the principles of safety, pertinence, practicability, and efficacy; choose exercise prescriptions suitable for individuals; and conduct sports and fitness activities under the guidance of professionals. Here, we summarize the different effects of various exercise modes on different groups of people. Based on the findings of this paper, we propose the following suggestions for the bone building needs of different populations. 1) Children and adolescents should be encouraged to participate in more intense speed and impact exercises to provide sufficient mechanical stimulation for their bones and to promote their growth and development. 2) For middle-aged and elderly individuals with acceptable physical status, menopausal women or patients with mild OP, an exercise mode combining resistance and impact training with appropriate intensity is the first choice to delay bone loss and prevent OP. In addition, rhythmic gymnastics and Tai Chi are also recommended activities for these individuals. 3) Low-load and multifrequency targeted resistance training is a relatively suitable exercise mode for older individuals or patients with other serious basic diseases and poor physical condition. If conditions permit, jump training can also be added to improve balance and proprioception. 4) For some special populations, such as professional athletes in swimming or long-distance running, pilots and astronauts, the risk of bone loss is greatly increased due to the particularity of their occupations. Therefore, impact and resistance training are indispensable for those populations (Table 3).

**Table 3:** Recommended exercise types for different groups of people with bone rebuilding needs.

Populations	Recommended exercise
Children and adolescents	Speed and impact training
Middle-aged and elderly individuals with acceptable physical status, menopausal women, and patients with mild OP	Combining resistance and impact training, rhythmic gymnastics and Tai Chi
Older individuals or patients with other serious basic diseases and poor physical condition	Low-load and multifrequency targeted resistance training, jump training
Professional swimmers or long-distance runners, pilots and astronauts, etc.	Impact and resistance training

Oxidative stress is an important mechanism mediating exercise regulation of bone homeostasis. Regular moderate exercise can improve the body's antioxidant defense ability, inhibit excessive oxidative stress responses, promote the positive balance of bone metabolism, delay age-related bone loss and deterioration of bone microstructures, and have an effective prevention and treatment effect on OP caused by many factors. As the saying goes, every coin has two sides. Sports themselves are a double-edged sword. Excessive high-intensity exercise does not benefit bone health but induces a high level of oxidative stress in the body, with a negative impact on bone tissue. For example, although intense speed and impact training have excellent effects in promoting bone growth and development and maintaining BMD levels, they are accompanied by excessive accumulation of ROS and a significant increase in the risk of various acute and chronic sports injuries. In conclusion, exercise, as an economic, effective and safe means, plays an extremely important role in the field of exercise health. As long as it is used properly, we believe that it will make greater contributions to human health.
